# Impaired Collagen Biosynthesis and Cross‐linking in Aorta of Patients With Bicuspid Aortic Valve

**DOI:** 10.1161/JAHA.112.000034

**Published:** 2013-02-22

**Authors:** Dick Wågsäter, Valentina Paloschi, Roeland Hanemaaijer, Kjell Hultenby, Ruud A. Bank, Anders Franco‐Cereceda, Jan H. N. Lindeman, Per Eriksson

**Affiliations:** 1 Atherosclerosis Research Unit, Center for Molecular Medicine, Department of Medicine, Karolinska University hospital, Karolinska Institutet, Stockholm, Sweden (D., V.P., P.E.); 2 TNO Metabolic Health Research, Leiden, the Netherlands (R.H.); 3 Department of Laboratory Medicine, Karolinska Institutet, Stockholm, Sweden (K.H.); 4 Department of Medical Biology, University Medical Center Groningen, Groningen, the Netherlands (R.A.B.); 5 Cardiothoracic Surgery Unit, Department of Molecular Medicine and Surgery, Karolinska Institutet, Stockholm, Sweden (A.F.C.); 6 Department of Vascular Surgery, Leiden University Medical Center, Leiden, the Netherlands (J.N.L.)

**Keywords:** aneurysm, aorta, bicuspid, collagen, valve

## Abstract

**Background:**

Patients with bicuspid aortic valve (BAV) have an increased risk of developing ascending aortic aneurysm. In the present study, collagen homeostasis in nondilated and dilated aorta segments from patients with BAV was studied, with normal and dilated aortas from tricuspid aortic valve (TAV) patients as reference.

**Methods and Results:**

Ascending aortas from 56 patients were used for biochemical and morphological analyses of collagen. mRNA expression was analyzed in 109 patients. Collagen turnover rates were similar in nondilated and dilated aortas of BAV patients, showing that aneurysmal formation in BAV is, in contrast to TAV,* not* associated with an increased collagen turnover. However, BAV in general was associated with an increased aortic collagen turnover compared with nondilated aortas of TAV patients. Importantly, the ratio of hydroxylysyl pyridinoline (HP) to lysyl pyridinoline (LP), 2 distinct forms of collagen cross‐linking, was lower in dilated aortas from patients with BAV, which suggests that BAV is associated with a defect in the posttranslational collagen modification. This suggests a deficiency at the level of lysyl hydroxylase (*PLOD1*), which was confirmed by mRNA and protein analyses that showed reduced *PLOD1* expression but normal *lysyl oxidase* expression in dilated aortas from patients with BAV. This suggests that impaired collagen cross‐linking in BAV patients may be attributed to changes in the expression and/or activity of *PLOD1*.

**Conclusions:**

Our results demonstrate an impaired biosynthesis and posttranslational modification of collagen in aortas of patients with BAV, which may explain the increased aortic aneurysm formation in BAV patients.

## Introduction

A bicuspid aortic valve (BAV) is by far the most common malformation of the aortic valve and is believed to result from abnormal aortic cusp formation during valvulogenesis. Although BAV can present as an isolated valve disorder, a large proportion of BAV patients develop ascending aortic aneurysm or dissection. In fact, prevalence of aortic dilatation in patients with BAV without significant valve dysfunction has been estimated to be as high as 50% to 70%.^1^ As yet, the pathophysiological basis for this aneurysmal dilatation is still unclear. It has been suggested that the increased aneurysm susceptibility relates to hemodynamic changes that result from the malformed aortic valve. However, the high incidence of aortic dilatation in BAV patients without valve dysfunction and the observed aneurysm formation in BAV patients after aortic valve replacement^2^ suggest that the process is not a response to the hemodynamic consequences of the BAV^3–4^ but rather reflects a structural defect of the ascending aorta.

Previous biomechanical studies demonstrated an increased wall stiffness of the aortic root in BAV, suggesting that BAV is indeed associated with a change in the mechanical properties of the ascending aorta.^5–6^ The mechanical properties of the arterial wall are essentially determined by the structural proteins elastin and fibrillar collagen, being responsible for the elastic recoil and for the resilience of the vessel wall, respectively. Consequently it has been shown that aneurysmal dilatation and ultimate rupture relate to quantitative or qualitative changes in collagen structures.^7^ Such changes could arise from excessive collagen degradation as result of a localized proteolytic imbalance (eg, the abdominal aortic aneurysm), from impaired collagen deposition (eg, vascular‐type Ehlers‐Danlos), or theoretically from a combination of these 2. To date, the molecular basis for BAV‐associated aneurysms is still unclear. In this context, we hypothesized that intrinsic fragility of the aortas in BAV patients may be a result of impaired collagen homeostasis. Therefore, we have performed a systematic evaluation of collagen synthesis and cross‐linking in patients with BAV. Nondilated and dilated ascending aorta segments from patients were studied with use of a normal tricuspid aortic valve (TAV) as a reference.

## Material and Methods

### Patients

The Advanced Study of Aortic Pathology (ASAP study) has been described previously.^8^ The study includes consecutive patients with aortic valve and ascending aortic disease undergoing elective open‐heart surgery at the Cardiothoracic Surgery Unit, Karolinska University Hospital, Stockholm, Sweden. None of the patients had significant coronary artery disease according to coronary angiography, and Marfan syndrome patients were excluded from the study. Demographics of patients included in the present analyses are presented in Table 1 (previously presented^9^). Maximal diameter was measured by transesophageal echocardiography as described previously.^8^ The ascending aorta was considered to be nondilated at a diameter of ≤40 mm and dilated at a diameter of ≥45 mm; subjects with aortic dimensions of 41 to 44 mm were not included in the analyses. A total of 109 patients (80 men and 29 women, mean age 61 years, age range 29 to 82 years) who were referred for elective aortic valve surgery and/or ascending thoracic aortic surgery were included. In total, the ascending aorta was dilated in 68 patients (52±5.4 mm [mean±SD]) and normal^10^ in 41 patients (35±3.6 mm).

**Table 1.** Patient Demographics

Aortic Diameter	BAV, mm		TAV, mm	
	≤40	≥45	≤40	≥45
No.	24	45	17	23
Sex, No. of women	5	10	5	9
Age, y*	56±10	60±12	69±11	61±15
BSA, m^2^	1.99±0.21	2.02±0.20	1.99±0.19	1.97±0.25
Maximum aortic diameter, mm* (minimim, maximum)	36.1±3.1 (30.2, 39.9)	50.3±3.3 (45.1, 57.1)	33.7±3.9 (27.5, 39.8)	53.8±7.6 (45.0, 70.0)
AS*	13	20	12	1
AR	11	14	5	18
Normal aortic valve	0	11	0	4

Values are presented as mean±SD or frequency. The patient demographics of the study have been described previously.^9^ BAV indicates bicuspid aortic valve; TAV, tricuspid aortic valve; BSA, body surface area; AS, aortic stenosis; AR, aortic regurgitation.

* ANOVA, *P*<0.01; TAV nondilated group older than the other groups.

* ANOVA, *P*<0.0001.

* Fisher exact test, *P*<0.01.

A BAV was present in 69 patients. Twenty‐four of these patients had a nondilated aorta (aortic diameter 36±3.1 mm, 19 men and 5 women, 16 with aortic valve stenosis and 8 with aortic valve regurgitation). Forty‐five of the patients with BAV had a dilated aorta (aortic diameter 50±3.3 mm, 35 men and 10 women, 31 with aortic valve stenosis, 11 with aortic valve regurgitation, and 3 with normal valves). A tricuspid aortic valve (TAV) was present in 40 patients. Seventeen of these had a nondilated aorta (aortic diameter 34±3.9 mm, 12 men and 5 women, 12 with aortic valve stenosis and 5 with aortic valve regurgitation). Twenty‐three of the patients with TAV had a dilated aorta (aortic diameter 54±7.6 mm, 14 men and 9 women, 1 with aortic valve stenosis, 19 with aortic valve regurgitation, and 3 with normal valves).

All aortic biopsy samples were taken from the anterior (convex) part of the aorta (ie, the site of aortotomy a few centimeters above the aortic valve). This study was approved by the Ethics Committee at the Karolinska Institutet, and patients were included after providing informed, written, and signed consent.

### Collagen and Cross‐linking Analyses

Cross‐sectional 10‐μm slices of paraffin‐embedded aortic tissue including the whole aortic wall were available from 56 patients (BAV nondilated, n=12; BAV dilated, n=16; TAV nondilated, n=15; TAV dilated, n=13). The sections were deparaffinized in xylene and hydrolyzed (110°C, 24 hours) in 1 mL of 6 mol/L HCl in 5‐mL polytetrafluoroethylene‐sealed glass tubes for analyses of collagen, pentosidine, proline, hydroxyproline, hydroxylysyl, HP, and LP content as described previously.^11^ Briefly, the samples were dried and redissolved in 1 mL of water containing 10 μmol/L pyridoxine (internal standard for the cross‐links HP and LP) and 2.4 mmol/L homoarginine (internal standard for amino acids) (Sigma‐Aldrich). Samples were diluted 5‐fold with 0.5% (vol/vol) heptafluorobutyric acid (Sigma‐Aldrich) in 10% (vol/vol) acetonitrile for cross‐link analysis, and aliquots of the 5‐fold diluted sample were diluted 50‐fold with 0.1 mol/L sodium borate buffer (pH 8.0) for amino acid analysis. Derivatization of the amino acids with 9‐fluorenylmethyl chloroformate and reversed‐phase high‐performance liquid chromatography of amino acids and cross‐links was performed on a 150 mm×4.6 mm Micropak ODS‐80TM column (Varian).

### Picrosirius Red Staining

Picrosirius red staining was used for the assessment of collagen fibers in aortas.^12^ Formaldehyde‐fixed 10‐μm sections of aortas from nondilated and dilated BAV and TAV were stained for 1 hour in saturated picric acid containing 0.1% picrosirius red. All sections were analyzed under linear polarized light at ×40 magnification, and images were captured with a Leica DC480 color video camera as previously described.^13^ Thick mature, tightly packed, and better‐aligned collagen fibers were orange‐red, and thin immature fibers were green.^14–15^ The total collagen content of lesions and the content of orange‐red and green fibers were quantified using Leica QWin and calculated as the ratio of chromogen area to lesion area.

### Gene Expression Analysis

Intima‐media and adventitia tissue samples were homogenized with FastPrep using Lysing Matrix D tubes (MP Biomedicals). Total RNA was isolated from 109 patients using TriZOL (Invitrogen, Paisley) and RNeasy Mini kit (Qiagen) as a cleanup including treatment with DNase. RNA quality was analyzed with an Agilent 2100 bioanalyzer (Agilent Technologies, Inc) (mean±SD RNA integrity numbers 7.0±0.6) and quantified with a NanoDrop (NanoDrop Products).

The RNA samples were hybridized and scanned at the Karolinska Institute microarray core facility. Affymetrix GeneChip Human Exon 1.0 ST arrays and protocols were used. For probe set– and meta probe set–level investigations (ie, the genomewide and regional investigations), CEL files were preprocessed using robust multichip average normalization as implemented in the Affymetrix Power Tools 1.10.2 package apt‐probeset‐summarize and log2 transformed. All investigations were done on the core set of meta probes provided by Affymetrix as published previously in the ASAP study.^16^

### Protein Preparation and Western Blotting

Medial layers from dilated aortas of BAV and TAV patients were finely minced and placed in a cell lysis buffer (2D Protein Extraction Buffer V [GE Healthcare] containing 30 mmol/L Tris buffer, pH 8.0) including a protease inhibitor cocktail (Roche Complete Mini EDTA Free). The tissue samples were mechanically homogenized using the TissueLyser system (Qiagen) according to manufacturer's instructions. The samples were subsequently sonicated in a water bath sonicator at 4°C (30 seconds' sonication at high intensity followed by 30 seconds' resting for a total of 8 minutes) and then pelleted in a microcentrifuge at 12 000*g* for 10 minutes at 4°C. The protein contents in the supernatant were measured with use of the Bradford protein assay (BioRad). Five micrograms of the protein lysates was resuspended in running buffer (BioRad) and subjected to SDS‐PAGE with 4% to 12% Bis‐Tris gels (Invitrogen NuPAGE). Electrophoresis was performed for 2 hours at 100 V. Western transfer to polyvinylidene difluoride membranes (Amersham Pharmacia Biotech), blocking in 3% BSA TTBS, and incubation with antibodies were performed according to standard protocols. The blot was incubated with primary polyclonal antibody against *PLOD1* (Santa Cruz Biotechnology) overnight and then for 1 hour with horseradish peroxidase–labeled secondary antibody, which was detected with enhanced chemiluminescence Western blot detection reagent (Amersham, GE Healthcare).

### Immunohistochemistry

Paraffin‐embedded sections from dilated and nondilated aortas of tricuspid and bicuspid biopsies were used. To unmask protein binding sites, sections were treated with DIVA Decloaker (Biocare Medical) solution in boiling water. Endogenous peroxidase activity was quenched by treatment with 3% hydrogen peroxide for 5 minutes followed by incubation with goat normal serum (1:5 in PBS) (Vector). Sections were then incubated with *PLOD1* primary antibody (Abgent Nordic Biosite) at 4°C overnight. Subsequently, sections were incubated with secondary biotinylated anti‐rabbit IgG (Vector). Avidin‐biotin peroxidase complexes (Vector) were added, followed by visualization with 3,3′‐diaminobenzidine tetrahydrochloride (Dako). All sections were counterstained with Mayer hematoxylin (Histolab Products).

### Masson Trichrome Staining

Masson trichrome method (HT15‐1KT kit; Sigma‐Aldrich) was used to detect collagen fibers in paraffin‐embedded aortic sections. Staining was performed according to the manufacturer's instructions, resulting in light blue color for collagen and red for muscle fibers and cytoplasm.

### Electron Microscopy

For scanning electron microscopy (SEM), aortic biopsy samples were fixed by immersion in 2% glutaraldehyde and 1% paraformaldehyde in 0.1 mol/L PBS (pH 7.4). The specimens were briefly rinsed in distilled water and placed in 70% ethanol for 10 minutes, 95% ethanol for 10 minutes, absolute ethanol for 15 minutes at room temperature, and pure acetone for 10 minutes and then dried. Specimens were then dried using a critical point dryer (CPD 010, Balzer) using carbon dioxide. After drying, specimens were mounted on an aluminum stub and coated with carbon (MED 010, Bal‐Tec). The specimens were analyzed in an Ultra 55 field emission scanning electron microscope (Zeiss) at 3 kV.^17^

For transmission electron microscopy (TEM), aortic tissues were fixed by immersion in 2% glutaraldehyde and 1% paraformaldehyde in 0.1 mol/L PBS (pH 7.4), rinsed in the same buffer, and postfixed in 2% osmium tetroxide. After dehydration, the samples were embedded in LX‐112 (Ladd Research Industries Inc). Semithin sections were cut and stained with toluidine blue O and used for light microscopic analysis. Ultrathin sections (≈40 to 50 nm) were cut by a Leica ultracut UCT (Leica), contrasted with uranyl acetate followed by lead citrate, and examined in a Tecnai 12 Spirit Bio TWIN transmission electron microscope (FEI Company) at 100 kV. Digital images were taken by using a Veleta camera (Olympus Soft Imaging Solutions GmbH).^18^

To determine the diameter of fibers, areas with transverse‐sectioned fibers were selected. Measurement was performed on captured digital images using microscope software (Olympus Soft Imaging Solutions, GmbH).^19^ A pilot study was performed using cumulative mean plot to determine the number of fibers needed for an appropriate measurement.

### Statistical Analysis

The statistical analysis was performed with StatView for Windows software (release 5.0.1, SAS Institute, Inc). Comparisons were done with ANCOVA with Scheffé test for posthoc comparisons. All the binary variables were tested with Fisher exact test, and linear regression was used to compare different groups while adjusting for age. Statistical analyses in Figures 3 and 5 were performed with a Mann–Whitney *U* test. *P* values of <0.05 were considered significant, and skewed data were log transformed.

## Results

### Lower Collagen Turnover in Dilated Aorta of BAV Compared With TAV Patients

**Figure 1.**
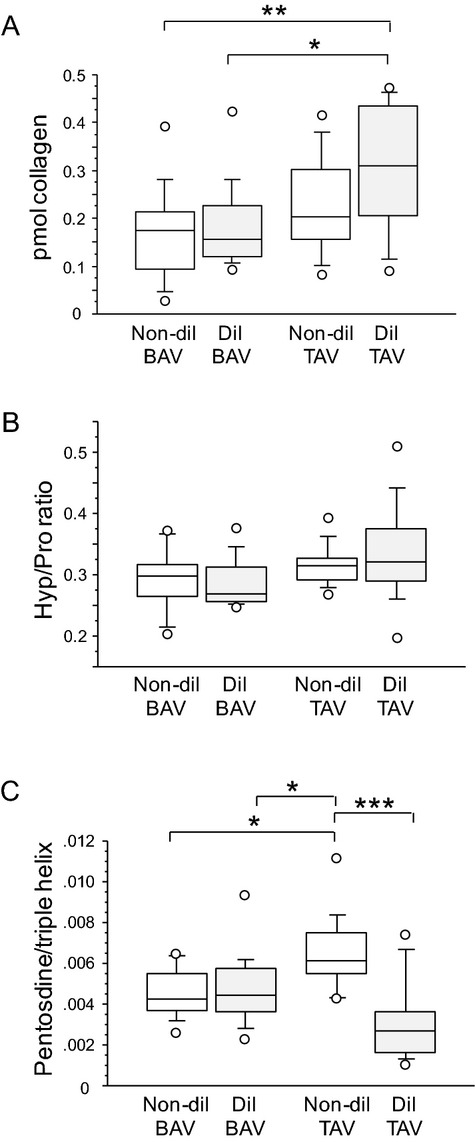
Collagen content (A, B) and turnover (C) in nondilated and dilated aortas from patients with BAV and TAV. *P<0.05, **P<0.01, ***P<0.001 after adjustment for age. BAV nondilated (Non‐dil), n=12; BAV dilated (Dil), n=16; TAV nondilated, n=15; TAV dilated, n=13. BAV indicates bicuspid aortic valve; TAV, tricuspid aortic valve; Hyp/Pro, hydroxyproline/proline.

**Figure 2.**
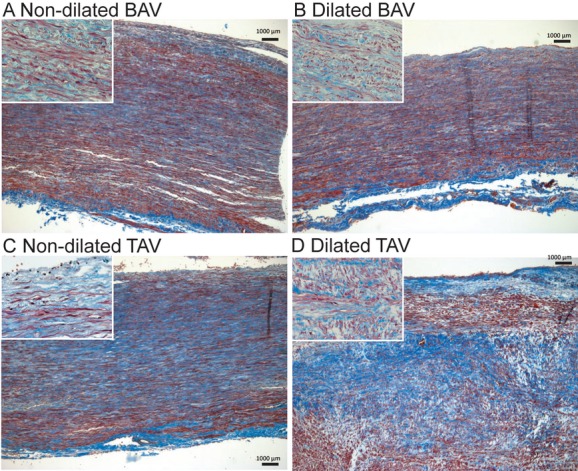
Masson trichrome staining of dilated and nondilated aortas from patients with BAV and TAV. Scale bar=1000 μm. Representative photomicrographs from 16 patients in total are shown. BAV indicates bicuspid aortic valve; TAV, tricuspid aortic valve.

High‐performance liquid chromatography–based biochemical evaluation of collagen in ascending thoracic aortas showed similar overall collagen concentrations in nondilated aortas of BAV and TAV patients (Figure 1A and 1B). Furthermore, there were no differences in mRNA expression of procollagens in nondilated aortas between TAV and BAV patients (Table 2). The extent of collagen turnover was assessed by the relative expression of pentosidine, a marker of nonenzymatic glycation. Under normoglycemic conditions, collagen glycation is largely determined by the half‐life of collagen (ie, a high relative pentosidine concentration reflects a low collagen turnover). The validity of this approach is shown by the results in TAV patients with aneurysm, which showed a sharply decreased half‐life, indicating increased collagen turnover in these patients (Figure 1C). In contrast, similar collagen turnover rates were found in nondilated and dilated aortas of BAV patients, showing that aneurysm formation in BAV is *not* associated with an increase in collagen turnover. In line with this, clear fibrotic changes (Masson trichrome staining) were observed in dilated aortas of TAV, but not BAV, patients (Figure 2).

**Table 2.** Intima‐Media mRNA Expression of Collagens in Ascending Aortas From BAV and TAV Patients

Transcript	BAV Nondilated, Mean±SD	BAV Dilated, Mean±SD	*P*‐Value (BAV Nondilated vs Dilated)	TAV Nondilated, Mean±SD	TAV Dilated, Mean±SD	*P* Value (TAV Nondilated vs Dilated)	*P* Value (BAV vs TAV in Nondilated)	*P*‐Value (BAV vs TAV in Dilated)
No.	24	45		17	23			
*COL1A1*	9.65±0.48	9.97±0.60	NS	9.63±0.47	10.71±0.86	<0.001	NS	<0.001
*COL1A2*	11.85±0.40	11.96±0.35	NS	11.68±0.45	12.18±0.39	<0.01	NS	NS
*COL3A1*	10.73±0.41	10.68±0.45	NS	10.63±0.53	11.18±0.53	<0.01	NS	<0.01

All expression measurements were robust multichip average normalized and log2 transformed and expressed as arbitrary units. BAV indicates bicuspid aortic valve; TAV, tricuspid aortic valve; NS, not significant.

**Figure 3.**
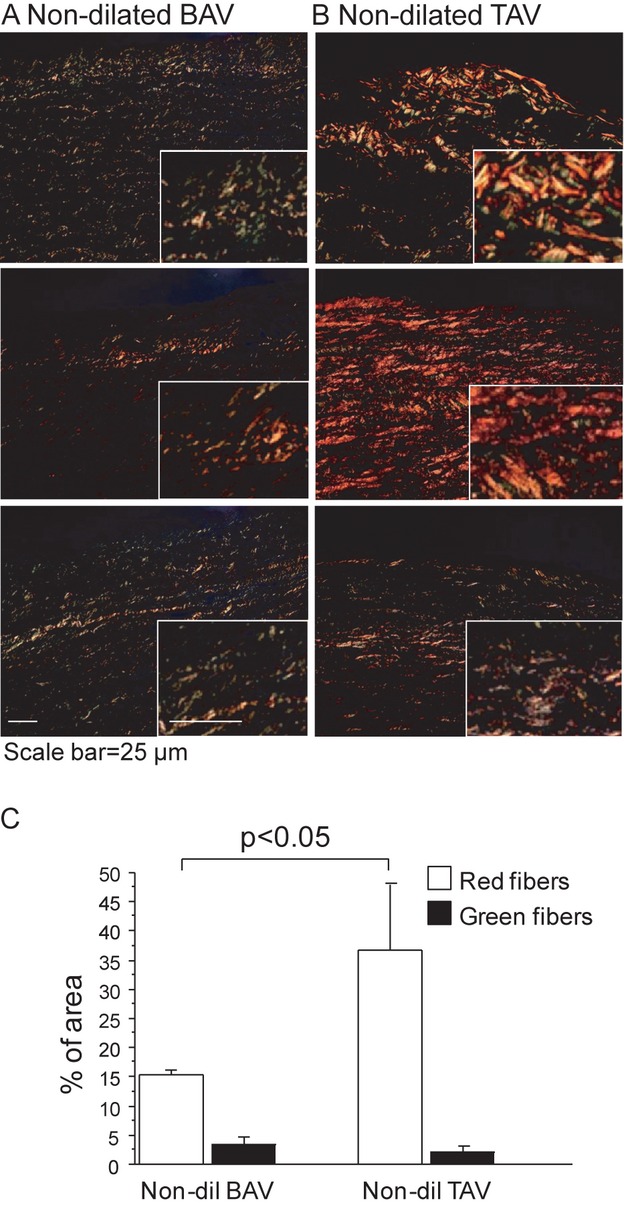
Picrosirius red staining of nondilated aortas from 3 randomly selected patients with BAV (A) and TAV (B). Green fibers indicate immature thin fibers and orange‐red fibers indicate mature, thicker, and better‐aligned collagen fibers. Magnifications of the stained areas are shown in right corner in each figure. All figures have been taken using the same conditions and settings. Scale bar=25 μm. C, Quantification of picrosirius red staining. Error bars are indicated by SEM. BAV nondilated (Non‐dil), n=3; TAV nondilated (Dil), n=3. BAV indicates bicuspid aortic valve; TAV, tricuspid aortic valve.

The generic lower levels of collagen glycation in nondilated BAV compared with nondilated TAV (Figure 1C) may suggest that BAV in general is associated with an increased aortic collagen turnover, even before the aneurysm formation. This notion was supported by picrosirius red staining under polarized light, which showed lower amounts of mature collagen in nondilated aortas from BAV patients (red in the image) than in TAV patients (Figure 3A through 3C).

**Figure 4.**
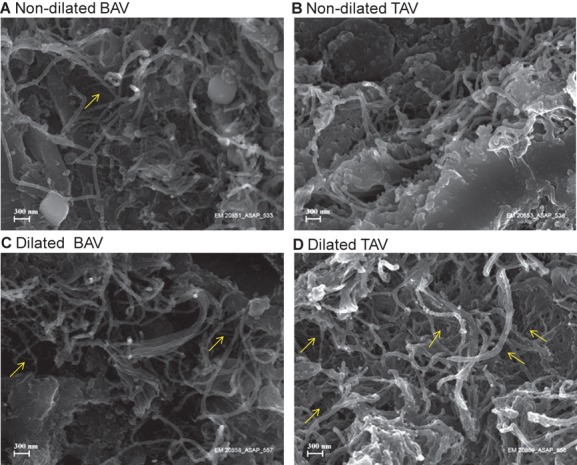
Scanning electron microscopy of collagen structure in ascending aortas. Nondilated aortas from patients with BAV (A) and TAV (B); dilated aortas of patients with BAV (C) and TAV (D). Representative photomicrographs from a total of 10 patients are shown. Thin collagen fibers are indicated in yellow. Bar=300 nm. BAV indicates bicuspid aortic valve; TAV, tricuspid aortic valve.

**Figure 5.**
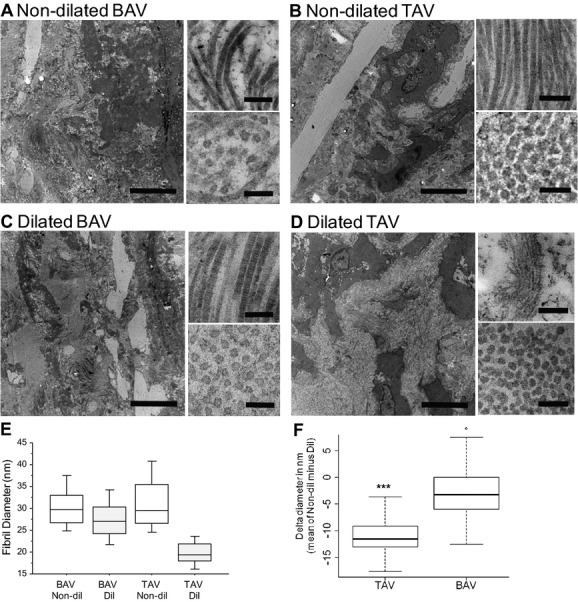
Transmission electron microscopy of collagen structure in ascending aortas. Nondilated (Non‐dil) aortas from patients with BAV (A) and TAV (B); dilated (Dil) aortas of patients with BAV (C) and TAV (D). Images represent overview, collagen fibers, and cross‐sectioned fibers. Bars are 5 μm, 200 nm, and 100 nm, respectively. Representative photomicrographs from a total of 4 patients are shown. E and F, Quantification of fibril diameters; 100 randomly selected fibrils from 4 patients were measured. ***P<0.001. BAV indicates bicuspid aortic valve; TAV, tricuspid aortic valve.

To further investigate the collagen structure in BAV and TAV aortas, we performed scanning electron microscopy and TEM, which supported the existence of a difference in the mechanism of aortic dilatation between BAV and TAV patients. An increased level of fibrosis was observed in dilated aortas of TAV, but not BAV, patients (an increased number of thin collagen fibers indicated by yellow arrows in Figure 4). This notion was confirmed by measuring the fibril diameter in TEM analysis (Figure 5E). Interestingly, the collagen network of BAV aortas appeared similar regardless of dilatation status (Figures 4A, 4B, 5A, and 5B).

### Impaired Posttranslational Modification of Collagen Associated With Aortic Dilatation in BAV Patients

**Figure 6.**
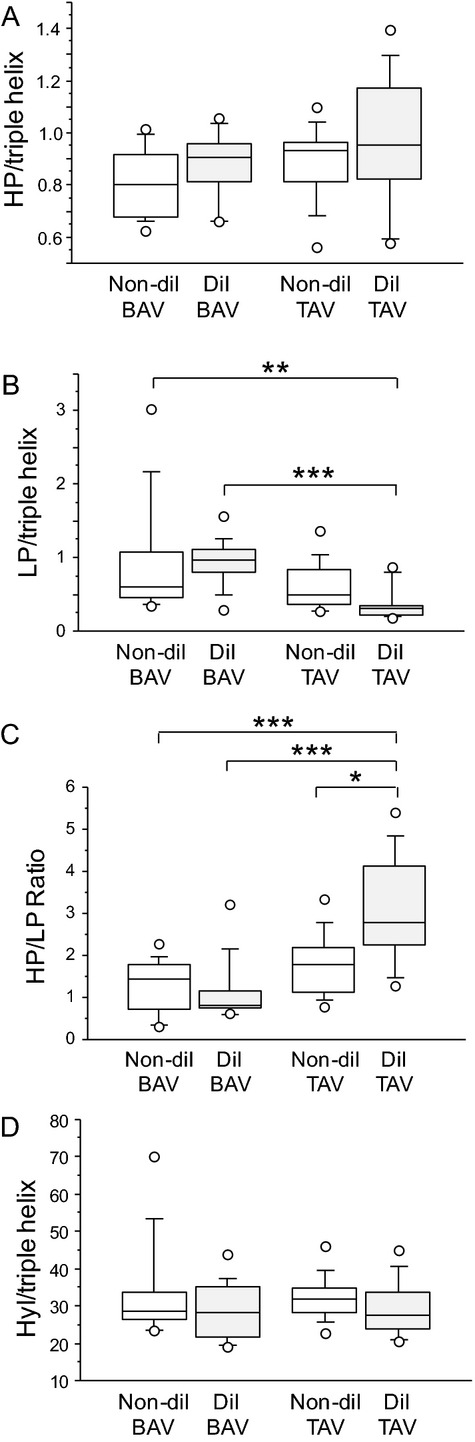
Cross‐linking of collagen in nondilated (Non‐dil) and dilated (Dil) aortas from patients with BAV and TAV. A, Hydroxylysylpyridinoline (HP); B, lysylpyridinoline (LP); C, HP:LP ratio; D, hydroxylysine (Hyl) residues. *P=0.05, **P<0.01, ***P<0.001 after adjustment for age. BAV nondilated, n=12; BAV dilated, n=16; TAV nondilated, n=15; TAV dilated, n=13. BAV indicates bicuspid aortic valve; TAV, tricuspid aortic valve.

The amount of stable mature cross‐links, as represented by HP and LP, was measured in nondilated and dilated ascending aortas of patients with BAV and TAV (Figure 6). Importantly, the HP:LP ratio was lower in dilated aortas of BAV patients compared with patients with TAV, which suggests that BAV is associated with a defect in the posttranslational modification of collagen (Figure 6C).

To further analyze the differences in cross‐linking of collagen fibrils in dilated ascending aortas in patients with BAV, we analyzed the amount of hydroxylysine residues. Hydroxylysine residues are essential for the formation of pyridinoline cross‐links in collagen fibrils. As shown in Figure 6D, the amount of hydroxylysine residues per collagen molecule did not differ between patients with BAV or TAV.

**Figure 7.**
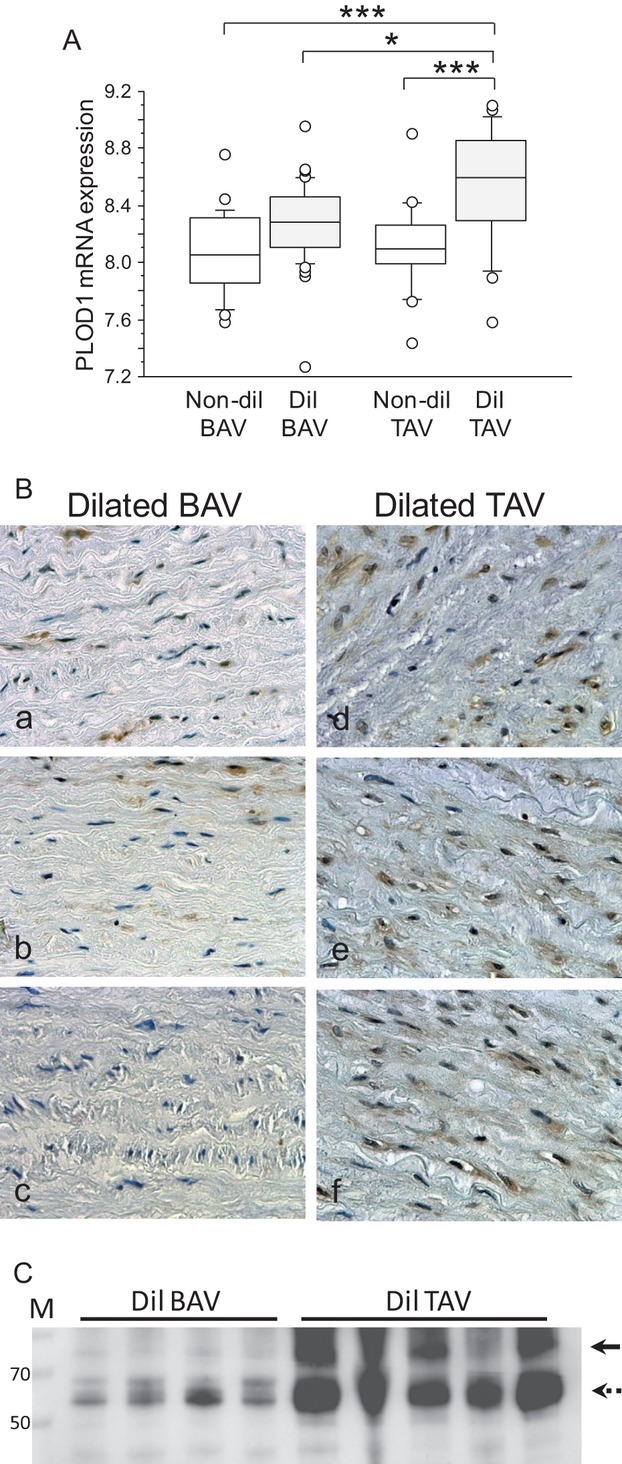
Decreased PLOD*1* expression in dilated aortas of BAV patients. A, mRNA expression of PLOD1 in intima‐media of ascending aortas from patients with BAV and TAV. BAV nondilated (Non‐dil), n=24; BAV dilated (Dil), n=45; TAV nondilated, n=17; TAV dilated, n=23. *P<0.05, ***P<0.001 after adjustment for age. B, PLOD*1* expression in media region of dilated ascending aortas from BAV (n=3) and TAV (n=3). C, Western blot of *PLOD1* using protein extracts from dilated ascending aortas from BAV (N=4) and TAV (N=5). Arrow denotes PLOD1 protein with molecular weight of 83 kDa; broken arrow shows upregulated band of lower molecular weight. BAV indicates bicuspid aortic valve; TAV, tricuspid aortic valve.

The lysyl hydroxylases PLOD1 and PLOD3 have been shown to convert lysine into hydroxylysine in the triple helix.^20–21^ The 2 single hydroxylsine residues in the triple helix that are involved in the formation of HP are specifically hydroxylated by *PLOD1*. The comparable overall levels of hydroxylysine in the triple helix, in combination with the decreased HP:LP ratio in dilated aortas of BAV compared with TAV patients, points to a specific decrease in the lysyl hydroxylation level of the 2 triple helical residues involved in HP formation. The lower HP:LP ratio in BAV suggests a decrease in *PLOD1* expression/activity in patients with BAV. As shown in Figure 6, *PLOD1* mRNA expression (Figure 7A) and PLOD1 protein expression (Figure 7B and 7C) were lower in intima‐media in dilated ascending aortas from patients with BAV than in patients with TAV. Immunohistochemical staining showed strong *PLOD1* expression in medial smooth muscle cells in dilated aortas of TAV patients, staining that was partially lacking in dilated aortas of BAV patients (Figure 7B). Expression of *PLOD1* in the adventitia did not differ in dilated aortas of BAV and TAV patients (data not shown). Similarly, *PLOD3* mRNA expression was lower in intima‐media but not in the adventitia of dilated aortas of BAV patients (data not shown).

**Figure 8.**
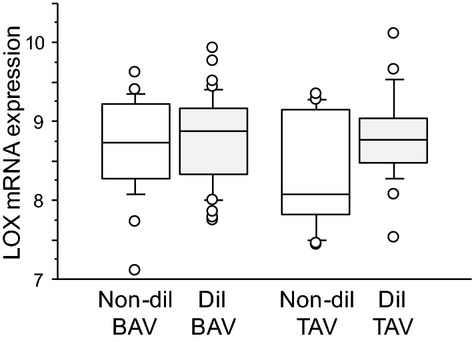
No difference in LOX mRNA expression between BAV and TAV. mRNA expression of LOX in intima/media of ascending aortas from patients with BAV and TAV. *P*>0.10 after adjustment for age. BAV nondilated (Non‐dil), n=24; BAV dilated (Dil), n=45; TAV nondilated, n=17; TAV dilated, n=23. BAV indicates bicuspid aortic valve; TAV, tricuspid aortic valve.

The final step in collagen formation is the cross‐link catalyzed by the rate‐limiting step of lysyl oxidase (LOX), thereby leading to the formation of stable collagen fibrils. As shown in Figure 8, there was no difference in mRNA expression of *LOX* in the intima‐media between BAV and TAV patients, thereby suggesting that the observed difference in cross‐linking is an effect of PLOD, and not LOX, activity.

## Discussion

In the present study, we have analyzed the structural basis for the increased aneurysm susceptibility in patients with BAV. We show that there are similar amounts of collagen in nondilated aortas of BAV and TAV patients. However, there are clear differences in collagen quality and turnover between the 2 patient groups. Aneurysm formation in patients with BAV appears to be associated with a qualitative collagen defect (ie, in the way in which collagen networks are structured). This is supported by clear differences in posttranslational modifications of collagen in BAV patients. Also, nondilated aortas of BAV patients demonstrate a higher collagen turnover than those of TAV patients, which suggests that BAV is associated with a generic defect in collagen stability. The latter is interesting because it has been shown that a majority of individuals with BAV will develop a dilated aorta later in life.^22^ As expected, aneurysm formation in patients with TAV was associated with an increased collagen turnover, probably reflecting the existence of a compensatory mechanism to balance enhanced degradation. This resulted in an increased production of newly synthesized collagen visualized with Masson trichrome staining. This finding is in line with previous data showing increased thickness of dilated aortas from TAV compared with BAV patients.^23^ The differences between BAV and TAV patients suggest fundamental differences in thoracic aortic aneurysm etiology that may, at least partially, explain the increased risk of aortopathy in patients with BAV.

Aneurysm formation relates to defects in the matrix structures responsible for the support of the vessel wall. Loss of medial elastin together with impaired collagen homeostasis is considered a hallmark of aneurysm formation.^24^ Although the loss of elastin will impair the elastic behavior, impaired collagen homeostasis underlies the actual dilation and the ultimate mechanical failure of the vessel wall. A major finding of the present study is the clear difference in collagen cross‐linking in patients with BAV and TAV with a lower HP:LP ratio in dilated aortas of BAV patients. This indicates that aneurysmal aortas of BAV patients may have reduced strength compared with the aortas of patients with TAV.

One of the key posttranslational modifications of collagen is carried out by *PLOD1* and *PLOD3*,^20^ the genes coding for the enzyme lysyl hydroxylase. PLOD converts triple helical lysyl residues to hydroxylysyl, which is one of the key modifications that is involved in cross‐link formation and collagen maturation. Mutations in *PLOD1* have been shown to be the cause of the kyphoscoliotic type of Ehlers‐Danlos syndrome,^20,25^ a syndrome associated with severe aortopathy including aneurysm and dissection. Furthermore, *Plod1*^−/−^ mice have a decreased aortic HP:P ratio and an increased risk of aortic rupture compared with wild‐type mice.^26^ Interestingly, previous studies have demonstrated that changes in the elastic properties of aortas from BAV patients result in increased wall stiffness of the aortic root.^5–6,27^

In the present study, we showed that PLOD mRNA and protein expression were lower in the medial layer of dilated aortas of BAV compared with TAV patients. LOX activity leads to the oxidation of lysine and hydroxylysine residues that form semialdehydes, which subsequently undergo spontaneous chemical reactions with LOX‐derived aldehydes or with unmodified lysine residues. LP is derived from 1 lysine and 2 hydroxylysines, and HP is derived from 3 hydroxylysines. However, in the present study, we could not detect any difference in medial mRNA expression of *LOX*. This suggests that the different HP:LP ratio between BAV and TAV patients is related to the observed changes in the expression of *PLOD1* and, therefore, the level of lysyl hydroxylation of the residues involved in HP and LP formation.

We could not detect a significant difference in the amount of hydroxylysine residues per collagen molecule between the groups. This is not surprising because a decrease of 1 to 2 residues compared with the total number of ~30 hydroxylysine residues per triple helix (Figure 6D) is not likely to be detected. However, a minor change in hydroxylysine residues is more likely to be indicated by the HP:LP ratio (Figure 6C).

Markedly lower pentosidine levels and increased expression of the collagen synthesis machinery in aneurysms in TAV patients suggest a shortened collagen half‐life, a finding that presumably reflects excess collagen degradation and compensatory collagen deposition, an observation that bears similarities to the abdominal aortic aneurysm in which excess proteolytic activity is matched by enhanced collagen deposition.^11^ However, the aortic dilation in BAV patients did not show the expected increase in collagen turnover that occurs in TAV patients.

Collagen content is not only dependent on synthesis and modification but also on degradation by collagenases. In fact, we could observe a decrease in pentosidine expression in aortas from patients with BAV compared with nondilated aortas of TAV patients, which indicates young collagen and a defect in collagen maturation. This was further confirmed by picrosirius red staining showing more mature collagens in nondilated aortas from TAV samples compared with aortas from BAV patients. In a recent study, we analyzed the expression of all known matrix metalloproteinases (MMPs) in dilated and nondilated ascending aortas of BAV and TAV patients.^9^ However, we could not detect any mRNA expression of the interstitial collagenases *MMP‐1* and *‐13* or of the neutrophils‐expressed *MMP‐8*. We and others have shown that ascending aortic aneurysm in patients with TAV, but not BAV, is associated with infiltration of inflammatory leukocytes.^16,28^ Accordingly, expression of *MMP‐9* and *cathepsin S,* 2 inflammation‐related proteases, were higher in dilated aortas from TAV compared with BAV patients (data not shown). Despite ample expression of the proteolytic enzymes, there was a higher expression of collagen in dilated aortas from TAV patients compared with BAV patients (Figure 1A and Table 2), which presumably reflects compensatory fibrotic changes as a result of the disease process. Instead, the dilated aorta of BAV patients is characterized by a lower level of mature collagen fibers and a lower collagen turnover, suggesting an intrinsic fragility of the aorta.

One limitation of the study is that we have too few samples to perform subgroup analysis to distinguish tissues derived from patients with aortic valve regurgitation and stenosis, which could help to further understand if interplay exists between collagen biosynthesis and hemodynamic forces.

Taken together, these data suggest that impairments in the structure and biosynthesis of collagen due to a lower activity of lysyl hydroxylases may be involved in the increased aneurysm susceptibility observed in patients with BAV.
